# Sulfate Alters the Competition Among Microbiome Members of Sediments Chronically Exposed to Asphalt

**DOI:** 10.3389/fmicb.2020.556793

**Published:** 2020-09-29

**Authors:** Antonios Michas, Mourad Harir, Marianna Lucio, Gisle Vestergaard, Anne Himmelberg, Philippe Schmitt-Kopplin, Tillmann Lueders, Dimitris G. Hatzinikolaou, Anne Schöler, Ralf Rabus, Michael Schloter

**Affiliations:** ^1^Research Unit Comparative Microbiome Analysis, Helmholtz Zentrum München, Helmholtz Association of German Research Centers, Neuherberg, Germany; ^2^Chair of Soil Science, Technical University of Munich, Freising-Weihenstephan, Germany; ^3^Research Unit Analytical BioGeoChemistry, Helmholtz Zentrum München, Helmholtz Association of German Research Centers, Neuherberg, Germany; ^4^Chair of Analytical Food Chemistry, Technical University of Munich, Freising-Weihenstephan, Germany; ^5^Department of Health Technology, Technical University of Denmark, Lyngby, Denmark; ^6^Institute of Groundwater Ecology, Helmholtz Zentrum München, Helmholtz Association of German Research Centers, Neuherberg, Germany; ^7^Department of Ecological Microbiology, University of Bayreuth, Bayreuth, Germany; ^8^Enzyme and Microbial Biotechnology Unit, Department of Biology, National and Kapodistrian University of Athens, Attica, Greece; ^9^Institute for Neuropathology, Charité University Hospital Berlin, Berlin, Germany; ^10^Institute for Chemistry and Biology of the Marine Environment, Carl von Ossietzky University of Oldenburg, Oldenburg, Germany

**Keywords:** sediment microbiomes, asphalt exposure, sulfate-reducing microorganisms, methanogens, competition, organic material

## Abstract

Sulfate-reducing microorganisms (SRMs) often compete with methanogens for common substrates. Due to thermodynamic reasons, SRMs should outcompete methanogens in the presence of sulfate. However, many studies have documented coexistence of these microbial groups in natural environments, suggesting that thermodynamics alone cannot explain the interactions among them. In this study, we investigated how SRMs compete with the established methanogenic communities in sediment from a long-term, electron acceptor-depleted, asphalt-exposed ecosystem and how they affect the composition of the organic material. We hypothesized that, upon addition of sulfate, SRMs (i) outcompete the methanogenic communities and (ii) markedly contribute to transformations of the organic material. We sampled sediments from the test and proximate control sites under anoxic conditions and incubated them in seawater medium with or without sulfate. Abundance and activity pattern of SRMs and methanogens, as well as the total prokaryotic community, were followed for 6 weeks by using qPCR targeting selected marker genes. Some of these genes were also subjected to amplicon sequencing to assess potential shifts in diversity patterns. Alterations of the organic material in the microcosms were determined by mass spectrometry. Our results indicate that the competition of SRMs with methanogens upon sulfate addition strongly depends on the environment studied and the starting microbiome composition. In the asphalt-free sediments (control), the availability of easily degradable organic material (mainly plant-derived) allows SRMs to use a larger variety of substrates, reducing interspecies competition with methanogens. In contrast, the abundant presence of recalcitrant compounds in the asphalt-exposed sediment was associated with a strong competition between SRMs and methanogens, ultimately detrimental for the latter. Our data underpin the importance of the quality of bioavailable organic materials in anoxic environments as a driver for microbial community structure and function.

## Introduction

Sulfate-reducing microorganisms are anaerobic prokaryotes that use dissimilatory sulfate reduction to conserve energy and sustain their growth. The transfer of electrons to sulfate (SO_4_^2–^) leads to sulfide (S^2–^) formation and yields considerably less energy compared to other energy-conserving pathways (Thauer et al., 1977). Despite their energetic disadvantage and accompanying low growth rates, SRMs have been detected in various environments and have a significant impact on the sulfur and carbon cycles (Rabus et al., 2015). An unexpectedly high diversity of SRMs was recently discovered in multiple ecosystems (Vigneron et al., 2018), particularly in marine sediments, where they contribute substantially to the oxidation of the organic material (Bowles et al., 2014). The broad nutritional versatility of SRMs covers H_2_ and CO_2_, acetate and many organic compounds (Oude Elferink et al., 1994; Muyzer and Stams, 2008; Rabus et al., 2015), including various aliphatic and aromatic hydrocarbons (Widdel and Rabus, 2001; Rabus et al., 2016).

Based on the concept of the “thermodynamic ladder” in geomicrobiology (Chapelle and Lovley, 1992), SRMs fail to grow in sediment zones where microbes with higher energy output (e.g., denitrifiers or iron reducers) compete for the same substrates. Likewise, in the presence of sulfate, SRMs are expected to outcompete methanogens in anoxic environments. They have a higher affinity for H_2_ and acetate (Oude Elferink et al., 1994; Omil et al., 1998), the main substrates for hydrogenotrophic and acetogenic methanogens, respectively, and a higher energy yield compared to methanogenesis (McInerney et al., 2009; Bethke et al., 2011). Several studies have indeed observed that increased sulfate concentrations and sulfate-reducing activity inhibit methanogenesis (Lovley et al., 1982; Lovley and Klug, 1983; Siegert et al., 2011; Hausmann et al., 2016; Ma et al., 2017). However, the outcome of the competition between SRMs and methanogens can differ depending on the environment. From mathematical models and experiments, Bethke et al. (2008, 2011) concluded that acetoclastic methanogenesis can be thermodynamically more favored than sulfate reduction. Nevertheless, methanogens are still outcompeted in bioreactor experiments, possibly due to their slow growth, when substrate concentrations are maintained low by the SRMs. This implies that thermodynamics alone cannot explain the interactions between these microbial groups in different environments; numerous factors, such as pH and total organic carbon (Visser et al., 1996; Mitterer, 2010), can affect the outcome of these interactions. In some experiments, coexistence of SRMs and methanogens or even higher methanogenic activity have been observed and attributed to the use of non-competitive substrates (Oremland and Polcin, 1982; Maltby et al., 2018) or differing ability to colonize the substrate matrix (Isa et al., 1986).

Natural changes in the environmental conditions should affect the outcome of the competition, by favoring the proliferation of different microbial groups. It is an open issue how SRMs compete for organic compounds with already well-established methanogenic communities, such as the ones in electron acceptor-depleted, oil-exposed ecosystems, after a sulfate increase. In order to study such a competition pattern in more detail, we took advantage of the natural presence of an asphalt oil spring at a land-to-sea transition fen ecosystem called Keri Lake, Greece. This spring results in a long-term impact of the asphalt oil on the surrounding sediment. A former metagenomic study compared the exposed sediments to a site with no oil impact in the vicinity of the spring, and showed that the persistent exposure to asphalt oil has favored the presence of syntrophic fermenting and methanogenic microbiota (Michas et al., 2017). Considering that sulfate is annually provided by the neighboring marine environment to both sites, by surface seawater surges or intrusion in groundwater, we investigated in this study the impact of sulfate addition on the microbiomes of sediment microcosms. Based on our previous observations, using sediments from the same sites, we compared the impact on (i) a diverse wetland community established in the presence of abundant plant material (peat) and electron acceptors (i.e., sulfate and nitrate) and (ii) a specialized community shaped under the presence of asphalt hydrocarbons and the absence of sulfate, with a higher potential for lower energy output metabolism. Our analysis focused on the abundance, activity and diversity of SRMs and methanogens using molecular approaches, including qPCR and barcoding of respective marker genes and correlated our data to the present organic material. We hypothesized that the competition of SRMs with other microbiome members is affected by the environment and the microbiome structure. Thus, the effect of sulfate addition was analyzed along two lines of queries: (i) will SRM be significantly favored in the asphalt-exposed sediment, due to their clear energetic advantage over the adapted methanogenic communities, and (ii) what will be their impact on the degradation of hydrocarbons and other organic material?

## Materials and Methods

### Study Site and Sampling Campaign

Sediment sampling from Keri Lake (37.685°N 20.831°E) was conducted with permissions from the National Focal Point to the Convention on Biological Diversity/Ministry of Environment, Energy and Climate Change, as well as from the National Marine Park of Zakynthos. The samples were collected from a non-exposed (N) and a highly exposed to asphalt (H) sites at Keri Lake in close proximity; the sites have been previously described by Michas et al. (2017). The two sites are located in the same area and have similar distances from the aquatic bodies of the lake and the sea side, but sediments from the H site are saturated with hydrocarbons. Thus, any difference in the physicochemical parameters of the sediments, that might affect the indigenous microbiomes, are linked to the presence of the asphalt. At the H site, the high concentration of asphalt compounds was accompanied by the depletion of sulfate and superimposed onto the presence of plant material that was observed at the N site.

For this study, about 3 kg of sediment from 0.4 to 0.6 m depth from each site was transferred into 2 l air tight glass bottles. To minimize exposure to oxygen, we kept flushing the bottles with N_2_ gas throughout the process, and later stored them for 2 weeks under anoxic and dark conditions at room temperature, to let the microbiota recover from the disturbance during sampling.

### Preparation of Anoxic Microcosms

All media were prepared as described by Widdel and Bak (1992), with several modifications. Sulfate-free saltwater medium was used to simulate seawater conditions, by mixing basal medium (21 g l^–1^ NaCl, 3 g l^–1^ MgCl_2_ × 6H_2_O) with trace element solution (FeCl_2_, ZnCl_2_, MnCl_2_, CoCl_2_, CuCl_2_, NiCl_2_, Na_2_MoO_4_, H_3_BO_3_, HCl), selenite-tungsten solution (Na_2_SeO_3_, Na_2_WO_4_, NaOH) and NaHCO_3_. Medium without additional sulfate was used for the control microcosms, while Na_2_SO_4_ (4 g l^–1^) was included for the sulfate treatment. Reduced conditions were maintained with Na_2_S (1.6 mM) and pH was adjusted to 7. No resazurin was added to avoid any possible toxic effects on microbial cells.

The dry weight of the sediments from each site was determined gravimetrically by incubating ∼5 g of sample at 60°C until constant weight (2 days). For the incubations, 6.66 g and 5.83 g of fresh sediment (corresponding to 3.5 g of dry sediment) from the N and H sites, respectively, were mixed with 3.5 ml of sulfate-free or sulfate-containing medium in 15 ml glass vials (height 100 mm, diameter 14 mm) in an anoxic chamber. This resulted in four incubation conditions: (i) NC (non-exposed sediment, without sulfate), (ii) NS (non-exposed sediment, with sulfate), (iii) HC (highly exposed sediment, without sulfate), and (iv) HS (highly exposed sediment, with sulfate). Per condition, 40 microcosms were prepared. The presence of O_2_ was monitored throughout the process using a digital oximeter GMH 3691 (Greisinger electronic GmbH, Germany); concentrations were maintained below the detection limit of the instrument. Vials were sealed with butyl septa and screw caps and stored in a horizontal position under continuous shaking (75 rpm) in the dark at room temperature. Four replicate microcosms per condition were sampled approximately every week (days 0, 9, 16, 23, 30, and 40); day 0 microcosms remained cooled immediately after medium addition, until the preparation of all microcosms (∼4-5 h). Sampling was performed as follows. Microcosms were centrifuged (700 × *g*; 1 min; 20°C) and 1 ml of the supernatant was sampled using N_2_-flushed sterile single-use syringes and needles (23G × 1 1/4″; 0.6 × 30 mm). The supernatants were centrifuged again (6000 × *g*; 2 min; 20°C) to pellet the remaining sediment particles and filtered through Millex-GP filter units (0.22 μm; Merck Millipore Ltd., Ireland). The filtered supernatants and the microcosms were stored at −80°C until further analysis. While sulfate was consumed quickly during the first 2 weeks, the microcosms were incubated for 6 weeks in total.

### Sulfate Measurements

Sulfate concentrations in the supernatants were measured by the turbidimetric method described by Tabatabai (1974). Briefly, the supernatants were mixed with barium-gelatin solution (BaCl_2_ × 2H_2_O, gelatin, HCl) and, after shaking for 10 min, the turbidity was measured in duplicates on a Victor microplate reader (Perkin Elmer Inc., United States) and compared to a calibration curve prepared from Na_2_SO_4_ standard solutions.

### Nucleic Acid Extraction

DNA and RNA were coextracted from the microcosms with a modified version of the protocol reported by Griffiths et al. (2000). A total of two grams of sediment per sample were transferred in Nucleospin Bead Tubes Type A (four tubes, each with 0.5 g sediment; Macherey-Nagel GmbH&Co., Germany) and were subjected to cell disruption on a Precellys 24 homogenisator (5 m s^–1^; 30 s; Bertin Technologies, France) with 0.5 ml of hexadecyltrimethyl ammonium bromide buffer (NaCl, K_2_HPO_4_, KH_2_PO_4_, β-mercaptoethanol) and equal volume of phenol:chloroform:isoamyl alcohol (25:24:1, v/v/v; Carl Roth GmbH+Co, Germany) per tube. After centrifugation (16100 × *g*; 5 min; 4°C), the lysing step was repeated with 0.3 ml of the above solutions. The combined supernatants from both steps were purified with an equal volume of chloroform:isoamylalcohol (24:1, v/v; Carl Roth GmbH+Co), followed by centrifugation (16100 × *g*; 5 min; 4°C). Precipitation of nucleic acids was conducted with an equal volume of polyethylene glycol 6000 (30%) in 1.6 M NaCl for 2 h at 4°C and subsequent centrifugation (20000 × *g*; 10 min; 4°C). The resulted pellet was washed with 1 ml of ice-cold ethanol (70%) and, after ethanol removal, air-dried for 10 min at room temperature. Nucleic acids were resuspended in 50 μl of H_2_O (DEPC-treated), 40 μl of which were used for the isolation of RNA and 10 μl were directly stored at −20°C and used for DNA-based analysis.

In order to obtain a pure RNA fraction, DNA was digested with the DNase Max^®^ Kit (MO BIO Laboratories Inc., United States), using 1 U of DNase Max Enzyme and incubation at 37°C for 10 min. The purity of the obtained RNA preparations was tested by PCR, with the primers 968f and 1401r targeting bacterial 16S rRNA gene (Nübel et al., 1996), and gel electrophoresis (2% agarose) with ethidium bromide staining; the absence of PCR bands confirmed that DNA was completely digested. The PCR reactions contained Taq Polymerase (2.5 U), 1 × Taq PCR buffer, 1.5 mM MgCl_2_, 0.2 mM dNTPs, 0.3% bovine serum albumin (BSA), 5% dimethyl sulfoxide (DMSO), 10 pmol of each primer and 2 μl of RNA template in a final volume of 50 μl. Residual DNA was denatured at 95°C for 5 min and amplified by 30 cycles of 94°C for 1 min, 58°C for 30 s and 72°C for 1 min, followed by final extension at 72°C for 10 min; absence of a DNA band on the gel confirmed that DNA was completely digested. Complementary DNA was immediately synthesized from the isolated RNA using the High Capacity cDNA Reverse Transcription Kit (Thermo Fisher Scientific Inc., United States) and stored at −20°C until further analysis.

### Real-Time PCR

We analyzed the abundance of marker genes and their transcripts for the total community (bacterial and archaeal 16S rRNA gene), sulfate reduction (dissimilatory sulfite reductase; *dsrB*), methanogenesis (methyl CoM reductase; *mcrA*), and the degradation of monoaromatics (6-oxocyclohex-1-ene-1-carbonyl-CoA hydrolase; *bamA*), using quantitative PCR (qPCR) on an ABI 7300 Real-Time PCR System (Thermo Fisher Scientific Inc.). All genes were amplified using 1 × Power SYBR Green PCR Master Mix (Life Technologies Ltd, United Kingdom) to a final volume of 25 μl. PCR reaction mixtures for bacterial and archaeal 16S rRNA genes additionally contained BSA (0.06%) and 5 pmol of each primer, for *dsrB* included MgCl_2_ (2.5 mM), BSA (0.06%) and 10 pmol of each primer, for *mcrA* contained Taq polymerase (0.75 U; Life Technologies, Germany), 1 × Taq PCR buffer, BSA (0.024%), 0.3 × Q-solution (Qiagen GmbH, Germany) and 6.25 pmol of each primer, and for *bamA* contained Taq polymerase (1 U; Life Technologies, Germany), 1 × Taq PCR buffer, BSA (0.06%) and 10 pmol of each primer. The primer sets and detailed PCR thermal profiles used can be found in Supplementary Table S1. Cloned plasmids containing the genes of interest were prepared using the ZeroBlunt TOPO kit (Invitrogen AG, United States), following the manufacturer’s protocol, and used as positive standards. Samples were diluted 1:100 to minimize PCR inhibition, as confirmed in pre-experiments using the same sediment samples (data not shown). Efficiency of the qPCRs was calculated based on the linear curve of the positive standards according to the formula *E* = 10^∧^(−1/*slope*) and was higher than 88% in all cases. Zero values indicate that no amplification was observed and, thus, no genes or transcripts were detected (detection limits: 873, 27, 9237, 225 and 8 gene copies g^–1^ slurry for bacterial 16S rRNA, archaeal 16S rRNA, *dsrB*, *mcrA* and *bamA*, respectively; 2523, 77, 4140, 84119, and 68 copies of transcripts g^–1^ slurry for bacterial 16S rRNA, archaeal 16S rRNA, *dsrB*, *mcrA* and *bamA*, respectively).

### Amplicon Sequencing

The diversity of total prokaryotes and microbial groups of interest was assessed using amplicon sequencing; the primer systems are described in Supplementary Table S1. All reactions were performed in duplicates with 1 × NEBNext High Fidelity Master Mix (New England BioLabs Ltd., United Kingdom) and 1-10 ng of DNA template in a final volume of 25 μl. PCR reaction mixtures for 16S rRNA gene additionally contained BSA (0.06%) and 5 pmol of each primer, for *dsrB* included MgCl_2_ (2.5 mM), BSA (0.06%) and 10 pmol of each primer, while for *mcrA* contained BSA (0.024%), 0.3 × Q-solution (Qiagen GmbH, Germany) and 6.25 pmol of each primer. Primers carried overhangs, which were used to incorporate the indices for Illumina sequencing. One positive and one non-template samples were included for each gene to test the amplification of contaminant DNA. Duplicate reactions were pooled prior to DNA purification with Agencourt AMPure XP beads (0.6 × sample volume; Beckman Coulter Inc., United States). The concentration of DNA was measured, and the absence of primer dimers was confirmed by analysis on a Fragment Analyzer Automated CE System (Advanced Analytical Technologies Inc., United States). Barcoded sequences and Illumina indices from the Nextera XT Index kit v2 (Illumina Inc., United States) were incorporated by PCR amplification with 1 × NEBNext High Fidelity Master Mix, 2.5 μl of each primer and 10 ng of DNA template, as follows: 98°C for 30 s, eight cycles of 98°C for 10 s, 55°C for 30 s and 72°C for 30 s, followed by 72°C for 5 min. Purification of amplicons and quantification of DNA were repeated as described above. The libraries were diluted to 4 nM and paired-end sequencing was performed on a MiSeq instrument using the MiSeq Reagent Kit v3 for 600 cycles (Illumina Inc.). All datasets have been deposited to NCBI SRA under the BioProject ID PRJNA561486.

### Bioinformatic Analysis

Low-quality bases (*Q*-score < 15) and adapter sequences were trimmed from both ends of the sequenced reads using AdapterRemoval v2.1.0 (Lindgreen et al., 2012); reads shorter than 50 bp were discarded. Datasets were subsequently analyzed using Qiime2 v2018.8.0^1^ (Caporaso et al., 2010). Paired reads were merged and chimeras were removed using the *qiime dada2 denoise-paired* command (DADA2 R package v1.3.4; Callahan et al., 2016) with default filtering parameters and N-terminal trimming of 10 bp. Based on the quality scores and the amplicon lengths, C-terminal trimming of the forward and reverse reads was adjusted per gene (240 and 180 for 16S rRNA and *dsrB* genes, 280 and 240 for *mcrA* gene). Two samples were excluded from the analysis, due to failed gene amplification. Amplicon sequence variants (ASVs) were inferred at the same step and taxonomy was assigned using the *feature-classifier classify-sklearn* command against reference sequences that match the used primers. The reference sequences were extracted from public databases and Qiime2 classifiers were trained using the *feature-classifier extract-reads* and *fit-classifier-naive-bayes* commands, respectively. For the analysis of 16S rRNA amplicons, the SILVA database (release 132; Quast et al., 2013) was used, while for *dsrB* and *mcrA* amplicons the respective nucleotide databases were downloaded from the FunGene website^2^ (v9.5; GenBank 226 as of 7/24/2018; Fish et al., 2013). The performance of the trained classifiers was tested using a selected number of representative sequences and the accuracy at the genus level was confirmed.

### Fourier-Transform Ion Cyclotron Resonance Mass Spectrometry (FT-ICR-MS)

For the analysis of the composition of the organic matter, a solid-phase extraction was performed as previously described by Fernández-Remolar et al. (2013), and the supernatants were used for FT-ICR-MS analysis. FT-ICR-MS was conducted on a solariX FT-ICR mass spectrometer (Bruker Daltonik GmbH, Germany) equipped with a 12 T superconducting magnet (Magnex Scientific Inc., GB) and an APOLLO II electrospray ionization source (Bruker Daltonik GmbH) in the negative ionization mode. The injections were performed using a microliter pump at a liquid flow rate of 120 ml h^–1^. Both sheath gas and curtain gas consisted of nitrogen. A source heater temperature of 200°C was maintained to ensure rapid solvent evaporation in the ionized droplets. Spectra were acquired with a time domain-sampling rate of four megawords and 500 scans were accumulated for each spectrum within a mass range of m/z 147.4 to 1400. The spectra were externally calibrated based on arginine clusters and systematically internally calibrated with appropriate reference mass list, reaching accuracy values lower than 100 ppb in routine day-to-day measurements. Data acquisition and handling were performed using Data Analysis Software (v4.1; Bruker Daltonics). Elemental formulas for each peak by a software tool written in-house (NETCALC) were calculated as described by Tziotis et al. (2011). Final formulas were generated and categorized into groups containing carbon, hydrogen and oxygen atoms depending on the presence or absence of nitrogen and/or sulfur (molecular compositions: CHO, CHNO, CHOS or CHNOS), to reconstruct the group-selective mass spectra. van Krevelen diagrams (van Krevelen, 1950) visualize the elemental ratios of unambiguously assigned molecular formulas.

### Statistical and Data Analysis

Data analysis and visualization were performed using the statistical program R v3.5.1 (R Core Team, 2015) and RStudio v1.1.456. Qiime2 output ASV tables and taxonomic data were imported into R using the qiime2R package (Bisanz, 2018). ASVs detected in both the respective non-template and positive controls per gene were considered as contaminants and were removed from the datasets. Additionally, ASVs that were not assigned to any taxonomic level were discarded and the datasets were rarefied to equal sequencing depth (44608, 10510, and 2984 reads for 16S rRNA, *dsrB*, and *mcrA* genes, respectively) using the *rrarefy* command of the vegan package (Oksanen et al., 2019). Rarefaction curves were calculated with the *rarecurve* command of the same package; curves reached a plateau indicating sufficient sequencing coverage (Supplementary Figure S1). Alpha diversity indices were calculated using the microbiome package (Lahti and Shetty, 2012/2017). Sample ordination was performed by principal coordinates analysis (PCoA) on the Bray-Curtis distances and ASVs assigned to the same genus were merged using the phyloseq package (McMurdie and Holmes, 2013). The results were displayed graphically with phyloseq, ggplot2 (Wickham, 2016), and cowplot (Wilke, 2019).

Significant differences in the concentrations of sulfate, gene abundances, diversity indices and the relative abundance of detected genera were tested by robust *t*-tests and ANOVAs on trimmed means using the *yuen*, *t1way* and *t2way* commands of the WRS2 package (Supplementary Table S2; Mair and Wilcox, 2019). Briefly, initial differences between the two sediments were detected by robust *t*-tests (*yuen*) on day 0. Changes due to the sulfate treatment were detected by 2-way robust ANOVAs (*t2way*) in each sediment separately and significant changes over time were tested by 1-way robust ANOVAs (*t1way*) in each condition. Differentially abundant genera among conditions were identified by multiple comparisons; *p*-values were adjusted by the Benjamini and Hochberg (1995) method. In all tests, the differences were considered significant when *p*-value was <0.05.

Association network analysis was performed using the CoNet v1.1.1.beta tool (Faust and Raes, 2016) on Cytoscape v3.7.2 (Shannon et al., 2003). Taxa below sum 120 and 12 occurrences per condition were discarded and relative abundances were calculated. Networks were inferred based on the 1000 top and bottom edges for each of the Pearson, Spearman, Bray and Kullback–Leibler correlation methods with 1000 iterations. Final *p*-values were computed during bootstrapping and adjusted with Benjamini–Hochberg correction for multiple testing.

The relations between the amplicon sequencing and mass spectrometry datasets were evaluated following the experimental design constrains (two factors: condition and time, with three replicates per time point). We built two different multilevel sparse partial least square discriminant analysis models (multilevel-sPLSDA), integrating the FT-ICR-MS data with 16S rRNA gene data firstly and with *dsrB* gene data secondly. The validation was set using leave-one-out cross validation; two valid components for both models were set. The importance of the variables was evaluated based on their contribution to the first valid component. The elaboration was done with RStudio v1.2.1335 package mixOmics (Lê Cao et al., 2011; Rohart et al., 2017; Lucio et al., 2019).

## Results

### Sulfate Consumption

**FIGURE 1:**
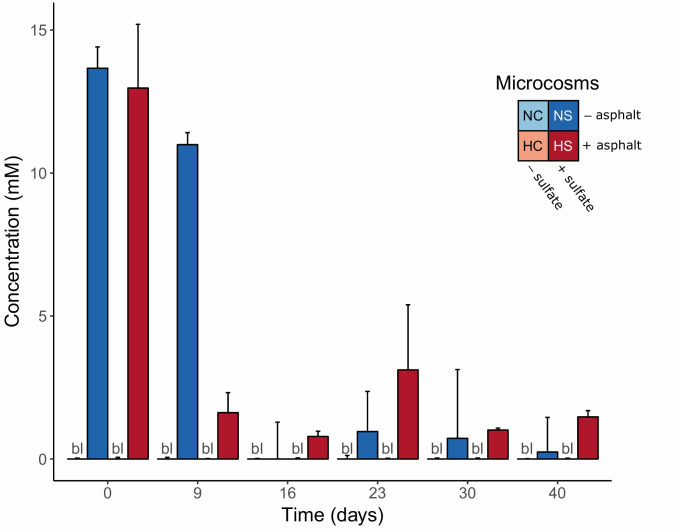
Sulfate concentrations in the supernatants of the anoxic microcosms over time. Error bars indicate the standard deviation of the measurements in the four replicate microcosms. bl = all replicates below detection limit.

Initial sulfate concentrations were approx. 13 mM in the sulfate-treated microcosms (NS and HS) and significantly decreased over time (*p*-value < 0.01, robust 1-way ANOVAs). In the HS microcosms, the concentrations decreased to <2 mM already after 9 days of incubation, whereas in the NS microcosms sulfate significantly dropped after 16 days (Figure 1). No sulfate was detected in the supernatants of the control microcosms (NC and HC; detection limit: 0.05 mg l^–1^). Based on these measurements, time points 0, 9, 16, and 40 days were selected for further molecular analysis, representing (i) the beginning of the experiment (day 0), (ii) the phase of sulfate consumption (days 9 and 16) and (iii) a period after sulfate consumption stopped (day 40).

### Abundance and Activity of Microbial Groups of the Sediment Microbiomes

**FIGURE 2:**
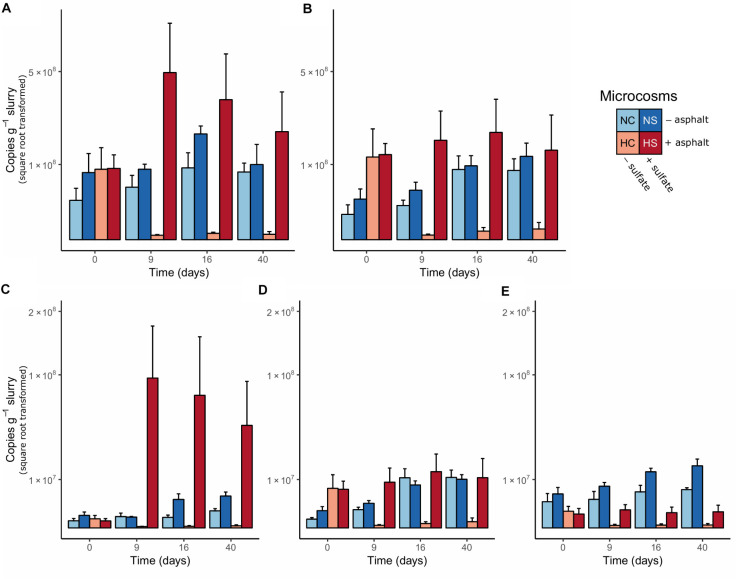
Copy numbers of **(A)** bacterial 16S rRNA, **(B)** archaeal 16S rRNA, **(C)** *dsrB*, **(D)** *mcrA*, and **(E)** *bamA* genes per g of dry anoxic sediment. The values are presented in square root transformed scale. Error bars indicate the standard deviation of the measurements in the four replicate microcosms.

Changes in key microbial groups (SRMs and methanogens), as well as total bacteria and archaea, were studied over time by the quantification of selected marker genes using the extracted DNA from the sediment samples (Figure 2). On day 0, the copy numbers of bacterial 16S rRNA and *dsrB* genes did not differ between the sediments. Only in the NC microcosms the bacterial 16S rRNA gene copy numbers were slightly lower, compared to the other conditions. The numbers of archaeal 16S rRNA and *mcrA* genes were significantly higher in the H microcosms (*p*-value < 0.01, robust *t*-test), while the number of *bamA* genes was significantly higher in the N microcosms (*p*-value < 0.01, robust *t*-test). The abundance of SRMs and methanogens was comparable on day 0 in the N microcosms, while in the H microcosms methanogens were higher in abundance compared to SRMs at the same time point. All detected genes gradually increased in abundance and peaked on day 16 in the N microcosms, with a higher increase of bacterial 16S rRNA, *dsrB* and *bamA* genes observed in the NS, compared to NC. Bacterial 16S rRNA and *dsrB* genes showed a stronger increase in the HS microcosms (3 and 5 times higher compared to NS, respectively) and peaked already on day 9, followed by a gradual decrease until the end of the experiment. Archaeal 16S rRNA and *mcrA* gene abundances did not change over time though. In the HC microcosms, the number of all genes (except for *dsrB*) declined significantly before the first 9 days (*p*-value < 0.05, robust 1-way ANOVA).

Activity patterns of the same microbial groups were analyzed by quantifying the number of transcripts in the extracted RNA samples (Supplementary Figure S2). On day 0, the number of all studied gene transcripts were higher in the N microcosms compared to H. However, the abundance of bacterial 16S rRNA and *dsrB* transcripts increased in the HS microcosms already on day 9, reaching the highest number of transcripts at this time point among all conditions, followed by a decrease toward day 40. The activity patterns in the HC microcosms did not change throughout the experiment and remained low. In the NS microcosms, a moderate increase in abundance of bacterial 16S rRNA transcripts was observed, which peaked on day 16, while in the NC no shifts were observed over time. The number of archaeal 16S rRNA transcripts was lower compared to bacteria and changed only slightly during the experiment in the N microcosms with a peak in activity on day 16. A slightly higher increase was observed in the NC compared to the NS. For *dsrB*, a clear effect of the sulfate addition was observed with an increased number of transcripts in the NS and HS microcosms, which peaked on day 16 and 9, respectively. In contrast, for *mcrA* transcripts, shifts mostly resulted from the origin of the sediments. During the experiment, an increase in the number of transcripts was observed in the N microcosms peaking on day 16, whereas only a moderate increase for *mcrA* transcripts was visible in the HS at the same time point and no response was observed for HC. Transcripts for *bamA* were below the detection limit for all samples analyzed.

### Community Structure of Microbial Groups of the Sediment Microbiomes

**FIGURE 3:**
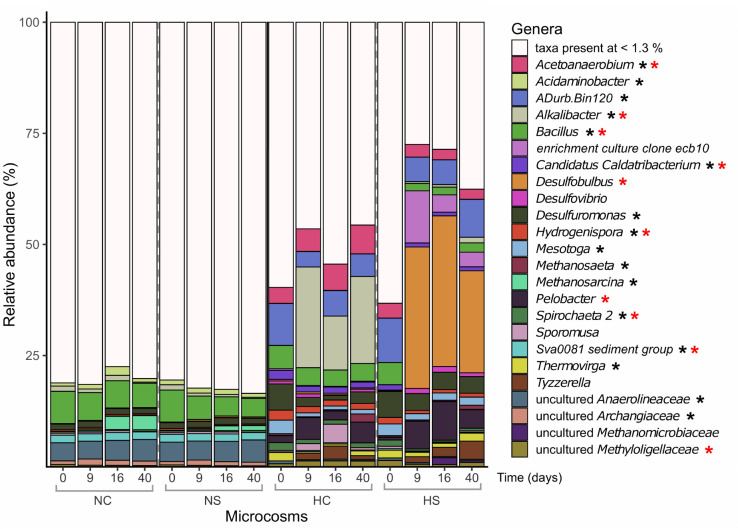
Relative abundance (%) of prokaryotic genera based on the taxonomic assignments of the amplified 16S rRNA genes. Only genera with abundance higher than 1.3% are shown. Differentially abundant genera in the different sediments on day 0 (robust *t*-test) are marked with a black asterisk. Genera that significantly changed over time in the N microcosms (2-way robust ANOVA) are marked with blue asterisks, while genera that significantly changed in the H microcosms (2-way robust ANOVA) are marked with red asterisks.

Prokaryotic α-diversity, based on 16S rRNA gene sequences, was significantly lower in the H microcosms compared to N at the beginning of the experiment (*p*-value < 0.01, robust *t*-test). It further decreased at later time points only in the H microcosms, with a stronger decrease in the HS (Supplementary Figure S3, top panel). A clear separation of the N and H microcosms was observed for β-diversity as indicated in the ordination plot (Supplementary Figure S4). Whereas NS and NC clustered close together throughout the experiment, HC and HS formed separate clusters during the incubation period already on day 9. Sediments from N harbored many low-abundance genera, with *Bacillus* and uncultured *Anaerolineaceae* dominating the community (Figure 3). A significant increase in *Methanosarcina* reads (*p*-value < 0.05, robust 2-way ANOVA) was observed over time, which was higher in the NC microcosms compared to NS. In the H microcosms, reads assigned to most highly abundant genera (e.g., *Chloroflexi ADurb.Bin120, Candidatus Caldatribacterium*, *Desulfuromonas*, *Hydrogenispora*, and *Mesotoga*) decreased over the incubation period and were replaced by *Alkalibacter* and *Desulfobulbus* in the HC and HS microcosms, respectively, already at early stages of the incubation experiment.

**FIGURE 4:**
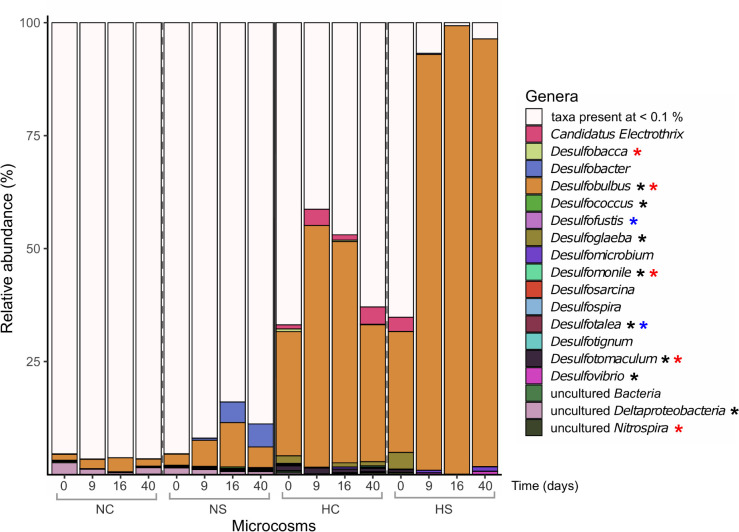
Relative abundance (%) of prokaryotic genera based on the taxonomic assignments of the amplified *dsrB* genes. Only genera with abundance higher than 0.1% are presented. Differentially abundant genera in the different sediments on day 0 (robust *t*-test) are marked with a black asterisk. Genera that significantly changed over time in the N microcosms (2-way robust ANOVA) are marked with blue asterisks, while genera that significantly changed in the H microcosms (2-way robust ANOVA) are marked with red asterisks.

The α-diversity of SRMs, as indicated by the analysis of the *dsrB* gene, was significantly higher in the N microcosms, compared to H, on day 0 (*p*-value < 0.01, robust *t*-test) and decreased at later time points under all conditions, with a significant effect found for NC and HS (*p*-value < 0.01, robust 1-way ANOVA; Supplementary Figure S3, middle panel). Overall, the taxonomic profiles of SRMs were impacted by the addition of sulfate in HS from day 9 on, based on β-diversity analysis (Supplementary Figure S5), whereas no clear separation could be observed for N after sulfate addition. The decrease in α-diversity in HC and HS was mainly linked to the strong dominance of *Desulfobulbus*. The reads assigned to this genus were already high at the start of the incubation period in both HC and HS (Figure 4) and increased over time. A stronger increase was observed in the HS compared to the HC microcosms. Interestingly, most *Desulfobulbus* 16S rRNA and *dsrB* gene ASVs were assigned to species *Desulfobulbus rhabdoformis* both in HC and HS. In contrast, most reads of amplified *dsrB* genes were assigned to uncultured SRMs in the N microcosms, with no clear information about their taxonomy.

**FIGURE 5:**
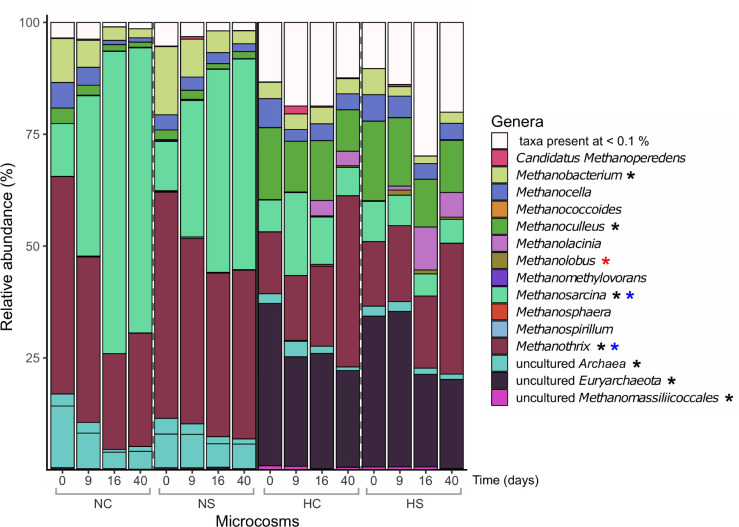
Relative abundance (%) of prokaryotic genera based on the taxonomic assignments of the amplified *mcrA* genes. Only genera with abundance higher than 0.1% are presented. Differentially abundant genera in the different sediments on day 0 (robust *t*-test) are marked with a black asterisk. Genera that significantly changed over time in the N microcosms (2-way robust ANOVA) are marked with blue asterisks, while genera that significantly changed in the H microcosms (2-way robust ANOVA) are marked with red asterisks.

The α-diversity of methanogens, as indicated by the *mcrA* gene, was comparable in all microcosms sampled on day 0 (Supplementary Figure S3, bottom panel). In the N microcosms, diversity significantly increased on day 9 (*p*-value < 0.03, robust 1-way ANOVA) and followed by a decrease until day 40, while in the H microcosms the same increase was not observed and diversity on day 40 was significantly lower compared to day 0 (*p*-value ≤ 0.01, robust 1-way ANOVA). Additionally, the addition of sulfate did not affect the β-diversity profiles of the H microcosms, while in NC and NS *mcrA* profiles clearly differed from day 9 on (Supplementary Figure S6), which is in contrast to the pattern observed for the 16S rRNA and *dsrB* genes. Over the incubation period, a lower increase in the number of reads assigned to the genus *Methanosarcina* was observed in the NS microcosms compared to NC, while reads associated with *Methanothrix* behaved the opposite (Figure 5). In the H microcosms, reads identified as *Methanothrix* increased over time along with a decrease in reads assigned to uncultured *Euryarchaeota*, but these changes were not significantly affected by the addition of sulfate.

Microbiome structure associations showed different patterns in each sediment. The addition of sulfate led to a slight increase of clustering coefficients in both sediments, but network centralization increased only in the H microcosms. The presence of *Desulfobulbus* showed a strong negative correlation to a big part of the established microbial community only in the HS microcosms, especially bacterial taxa (Supplementary Figure S7).

### Dissolved Organic Matter

**FIGURE 6:**
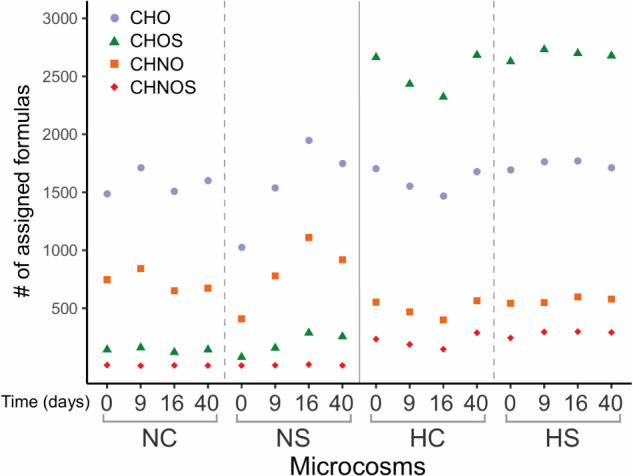
Number of assigned organic components of the different microcosms. Color-coded composition: blue, CHO; orange, CHNO; green, CHOS; and red, CHNOS.

Mass spectra of the N and H samples provided several thousand mass peaks, half of which could be assigned to molecular compositions CHO, CHNO, CHOS and CHNOS using conservative assignment criteria (Hertkorn et al., 2016; Handle et al., 2017). The patterns of these molecular compositions differed between the two sediment types (Supplementary Figure S8). The H microcosms showed a higher richness in heteroatoms content mainly composed of oxygen and sulfur compared to the N microcosms (Figure 6). This richness in *O,S*-functionalized compounds derives from the asphalt and its degradation organic fractions (i.e., biochemical alterations) in these sediments. Slightly higher numbers of CHO, CHOS and CHNO were observed on day 9 in the NC microcosms, compared to the remaining time points, while the same components increased in the NS from day 0 to day 16. All assigned molecules decreased in the HC microcosms until day 16 and increased toward day 40 but did not change over time when sulfate was added (HS) (Figure 6).

**FIGURE 7:**
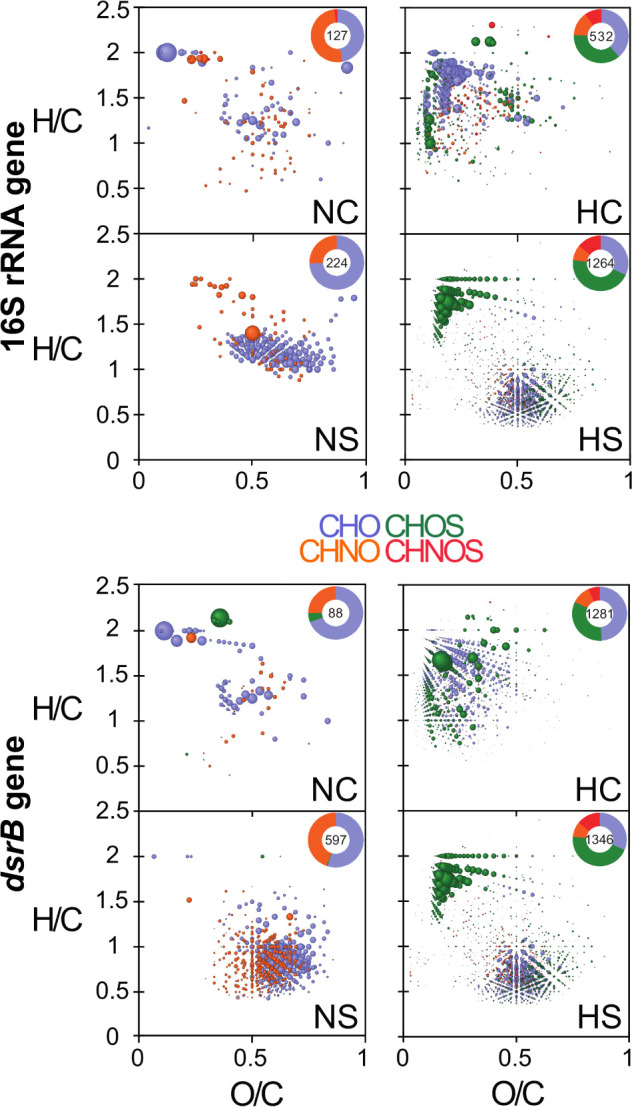
van Krevelen diagrams of the most correlated m/z values, based on the variables contribution on the first component. The diagrams visualize the chemical space of organic components in the microcosms containing non-exposed and highly exposed sediment, respectively (see also Supplementary Figure S10). Top panels are associated with the 16S rRNA gene, while bottom panels are associated with the *dsrB* gene. Insert histograms represent relative counts of components that are correlated with the respective genes. Color-coded composition: blue, CHO; orange, CHNO; green, CHOS; and red, CHNOS.

Multilevel sPLSDA models (Supplementary Figure S9) grouped homogeneous samples that share the same properties or trends of variables. Their examination indicated an overlap of groups NC and NS, which underlined a strong similarity of these classes. The other groups (i.e., HC and HS), placed in the negative coordinate system, denoted a negative correlation with NC and NS. This means that all variables that are increasing in one group are decreasing in the other. As output, each model gave a list of regression coefficients that, based on their value, determine the separation between the different groups. The coefficients could explain the correlations between masses and 16S rRNA taxa, as well as between masses and *dsrB* taxa (Lucio et al., 2019). Based on the highest coefficient values, the most important m/z (mass-to-charge ratio) variables per gene were assessed using van Krevelen plots. Therefore, we had one plot that expressed the correlations between m/z and 16S rRNA taxa, and one between m/z and *dsrB* taxa. The related components per gene in each condition are presented in Figure 7 and, in more detail, in Supplementary Figure S10.

M any of the assigned compounds were associated with changes in the diversity of 16S rRNA and *dsrB* genes. In the NC microcosms, organic compounds’ fingerprints associated with differentially abundant 16S rRNA and *dsrB* annotations were only slightly dissimilar (few saturated and oxygenated CHO and CHNO compositions) but considerably differed in the NS (Figure 7, left panels and Supplementary Figure S10), with differentially abundant *dsrB* taxa linked to increased CHO and CHNO compositions characteristic for lignin- and tannin-like compounds. These classes of refractory compounds have already been observed in soils and sediments (Derenne and Largeau, 2001). Unlike, the opposite was observed in the fingerprints of the H microcosms. Here the distribution of the molecular compositions showed almost no change in the HS but differed strongly in the HC microcosms (Figure 7, right panels and Supplementary Figure S10). Respectively, changes in the organic compositions were associated with most of the abundant 16S rRNA and *dsrB* genera, including *Alkalibacter* and *Desulfobulbus* (Supplementary Figure S11). Overall, the variance in the chemical compositions of the studied microcosms demonstrated the uniqueness of dissolved organic matter quality in these sediments in relation to the observed genera.

## Discussion

Several differences between the microbiomes of the proximate but distinct sediments were apparent from the beginning of the experiment. The non-exposed sediment harbored communities with higher total microbiome and SRM diversity, while the asphalt-exposed microbiomes contained higher abundance of total archaea and higher potential for methanogenesis, as expected according to our previous study (Michas et al., 2017). Transcription levels in the asphalt-exposed microcosms were significantly lower, which reveals the negative impact of the asphalt on the activity of sediment microbiomes.

The consumption of added sulfate coincided with an increase in the number of bacterial 16S rRNA and *dsrB* genes, indicating the growth of bacterial SRMs in both sediments. Even in the sulfate-depleted asphalt-exposed sediment of Keri Lake, SRMs were present and reduced sulfate upon its provision. Considering that some SRMs are capable of fermentation and acetogenesis (Muyzer and Stams, 2008; Rabus et al., 2015), and have been previously observed in sulfate-free co-cultures with methanogens (Bryant et al., 1977; Meckenstock, 1999), a seed bank of SRMs might survive in syntrophic associations with methanogens when sulfate is absent. Sulfate consumption and the increase in abundance of SRMs were faster in the asphalt-exposed sediment. This indicates that in hydrocarbon-saturated sediments under conditions favoring methanogenesis, fast-growing opportunistic species respond quickly and dominate the community if appropriate changes in electron acceptor availability occur. Similar to our observation, Vigneron et al. (2017) and James and Richardson (2020) observed a fast growth of SRMs after sulfate addition in an oil reservoir and a methanogenic bioreactor, respectively, underlining the ecological importance of these species during changing conditions.

Indeed, the main SRMs present in the asphalt-exposed microcosms already on day 0, were part of the highly versatile *Desulfobulbus* genus. Representative species of *Desulfobulbus* are capable of both respiratory and fermentative growth (Laanbroek et al., 1982; Sass et al., 2002; El Houari et al., 2017; Kharrat et al., 2017) and are considered generalists, based on their metabolic flexibility. Lineages within the *Desulfobulbaceae* family have been previously identified as carriers of genes coding for monoaromatic degrading enzymes (von Netzer et al., 2016), although the high share of *Desulfobulbus* did not result in an increased potential for degradation of monoaromatics as inferred via *bamA* gene abundance in our experiment. Members of the species *Desulfobulbus rhabdoformis*, found in the asphalt-exposed microcosms, is able to use small-molecular-weight compounds and has no vitamin needs, compared to other *Desulfobulbus* representatives (Lien et al., 1998). Its prevalence among SRMs already at the beginning of the experiment indicates that the asphalt exposure selects for sulfate reducers with narrow requirements for nutrients.

Despite the equal amount of sulfate added, higher abundances of SRMs were observed in the HS microcosms, compared to the NS. A slight increase in the potential and activity of methanogens was observed only when sulfate was low (day 16), indicating a strong competition between SRMs and methanogens. Since methanogens can only use a restricted range of substrates and thrive closer to the thermodynamic limit (Thauer et al., 1977; Liu and Whitman, 2008), this suggests that SRMs prefer more easily degradable substrates in the presence of recalcitrant asphalt compounds. Considering that hydrocarbon-degrading microorganisms thrive in the oil-water interphase (Pannekens et al., 2019), life is spatially restricted to specific niches in hydrocarbon-saturated environments, as has been shown for water droplets in the oil body itself (Meckenstock et al., 2014). Thus, the competing microbes are restricted to a limited space which probably intensifies the negative interactions among them. Methanogens are outcompeted due to the low energy yield of methanogenesis and slightly recover only after the number of SRMs decreases.

In contrast, the abundance of SRMs was less pronounced in the NS compared to the HS microcosms, suggesting that SRMs do not have the energetic advantage to outcompete the other members of a more diverse community, which doesn’t rely solely on low energy output metabolism. The addition of sulfate in the asphalt-free microcosms favored the growth of SRMs and inhibited methanogens only slightly, indicating that competition between them was weak, as it has been similarly observed in estuarine sediments (Sela-Adler et al., 2017). Unlike the asphalt-exposed microcosms, the potential for anaerobic degradation of monoaromatic compounds also increased and the presence of SRMs was associated with lignin- and tannin-like compounds. In this case, SRMs do not seem to focus on substrates that are shared with methanogens but rather exploit a wider range of organic compounds, e.g., plant-derived compounds that are more bioavailable when conditions are not saturated by asphalt, as observed previously too (Michas et al., 2017). It is also possible that the competition is minimized due to the presence of diverse substrates, as it has been observed in other organic-rich sediments (Holmer and Kristensen, 1994).

Interestingly, the impact of sulfate addition and growth of SRMs was reflected on the taxonomic profile of different microbial groups in each sediment. In the NS microcosms, among the detected methanogens, sulfate inhibited specifically the growth of *Methanosarcina*, the main methanogen that grew over time in all asphalt-free microcosms. *Methanosarcina* spp. are able to use several compounds via the known metabolic pathways of methanogenesis (Welander and Metcalf, 2005). It has been also reported that sulfate input can enhance the electron donor transfer from the methanogenic *Methanosarcina barkeri* to the sulfate-reducing *Desulfovibrio vulgaris* resulting in less energy output for the former (Phelps et al., 1985). Thus, SRMs possibly compete with and/or even scavenge electron equivalents directly from the *Methanosarcina*-associated consortia. In contrast, the structure of the methanogenic community did not change significantly in the HS microcosms, suggesting a negative feedback effect on all the detected methanogenic genera or that the 40 days period was not enough to observe a specific effect. Considering that growth of *D. rhabdoformis* has been observed on H_2_ and acetate (Lien et al., 1998), it is possible that these microbes compete with methanogens for both of these substrates. Interestingly, the growth of *Desulfobulbus* correlated negatively to the abundance of specific bacterial species that can potentially serve as fermenting partners for methanogens. Co-occurrence analysis should be interpreted with extreme caution though, considering that the networks were calculated based on relative but not absolute abundances. The inhibition of fermenters was confirmed by the communities observed in the HC microcosms; although the total potential and activity of microbiomes were significantly reduced, the presence of *Alkalibacter* was relatively enriched over time. *A. saccharofermentans*, the only known *Alkalibacter* representative, is an alkaliphilic organoheterotroph with the ability to ferment various sugars and has no vitamin requirements (Garnova et al., 2004), thus *Desulfobulbus* and *Alkalibacter* do not seem to directly compete for the same substrates. In this case, the negative impact might be attributed to the quick accumulation of sulfate reduction by-products (sulfite and sulfide), which might inhibit the syntrophic methanogenic consortia (Balderston and Payne, 1976; Karhadkar et al., 1987).

The competition between the microbiome members affected which microbial groups impacted the organic material in each sediment too. Sulfate reduction alters dissolved organic material in sediments (Chen et al., 2016, 2017; Luek et al., 2017), thus the changes in the organic matter compositions associated with the SRMs were compared to the changes associated with the total communities. In all asphalt-free microcosms, the fingerprints of SRMs overlapped only partly with the fingerprints of the total communities, indicating that other microbiome members contribute to changes in the available organic material as well. The addition of sulfate affected the increase of CHO and CHNO signatures, most likely through the production of new cellular compounds (i.e., optically active fluorescent compounds) by the growing SRMs (Chen et al., 2016; Luek et al., 2017), but the rest of the communities still played an important role in shaping the composition of the organic material. Available sulfates mostly ensure the anoxic transition between sulfate reduction and methanogenesis in sediments (Thang et al., 2013; Luek et al., 2017). Likewise, potential SRMs in the HC microcosms also affected the available organic material in the absence of sulfate. Despite their overall low abundance and activity, little changes in the amount of organic components were indeed observed. Recent studies have shown that sulfate reduction does not only occur in sulfate-rich sediment but also in sediments with low sulfate content (Holmkvist et al., 2011; Treude et al., 2014; Brunner et al., 2016; Glombitza et al., 2016). By comparing the organic components associated with changes in the abundance of total prokaryotes and SRMs, a preference of SRMs for CHOS components was revealed, which can be attributed to various environmental routes of reactions, including inorganic transformations and microbial degradation of source materials (Fukushima et al., 1992; Luek et al., 2017). This can also indicate a preference for alternative sulfur sources in the absence of sulfate, since the rest of the community was associated more with changes in CHO components. This was not the case in the HS microcosms though. The strong growth of *D. rhabdoformis* superimposed the rest of the community, underlining the dominant role of sulfate reducers in hydrocarbon-saturated environments and their ability to completely outcompete a big part of the established communities when sulfate is available. Overall, sulfate reduction and the associated microbiome members are the main factors responsible for the alteration and mineralization of organic materials in these extreme sedimentary systems. By comparing the organic components associated with changes in the abundance of total prokaryotes (16S rRNA gene) and sulfate reducers (*dsrB*), we observed that there is a preference of sulfate reducers for *S*-containing (CHOS) components.

## Conclusion

In the present study, we studied the competition of SRMs with other microbiome members in asphalt-exposed wetland sediments after sulfate input, which can be a result of seawater surges in land-to-sea transition ecosystems. We used sediments from sites non-exposed and highly exposed to asphalt at Keri Lake, Greece, which gave us the opportunity to compare two microbiomes that have been diversified over a long geological period due to natural selection under different conditions. Our results indicate that the competition between SRMs and methanogenic communities depends on the environment and the related microbiome, and has an impact on the organic material in the sediment. In the asphalt-free sediments, the bioavailability of degradable organic material (mainly plant-derived) allowed SRMs to use a variety of substrates, reducing interspecies competition with methanogens. In contrast, the SRMs in asphalt-exposed sediments were able to outcompete methanogens. Considering that methanogens are able to consume only small-molecular-weight compounds, we conclude that under these conditions SRMs also focus on these substrates, that are easily available compared to the lipophilic compounds of the asphalt. Ongoing genome assembly and metatranscriptomic analysis will provide further information about the metabolism of the key players in the microcosms, their potential substrates and the mechanistic background of the competition. The low number of transcripts though was a good indication that the microbial activity was not high in the microcosms. Further analysis of the microbial activity, by measuring the rates of sulfate reduction and methanogenesis as well as changes in pools of substrates and metabolic products (volatile fatty acids, CO_2_, CH_4_, SO_4_, and S_2_H), would give us a better picture about the response of the studied functional microbial groups.

## Data Availability Statement

The datasets presented in this study can be found in online repositories. The names of the repository/repositories and accession number(s) can be found below: https://www.ncbi.nlm.nih.gov/, PRJNA561486.

## Author Contributions

AM, AH, TL, DH, AS, RR and MS designed the study. AM collected samples, generated and analyzed qPCR data, generated sequencing data, and wrote manuscript. AM and AH prepared and sampled microcosms, and generated sulfate concentration data. AM and GV analyzed sequencing data. MH generated and analyzed mass spectrometry data. ML performed chemometric analysis for mass spectrometry data. AM, MH, RR, and MS conceptualized manuscript. All authors contributed to revisions and approved the final manuscript.

## Conflict of Interest

The authors declare that the research was conducted in the absence of any commercial or financial relationships that could be construed as a potential conflict of interest.

## Footnotes

1 https://qiime2.org/

2 http://fungene.cme.msu.edu/

## Abbreviations

ASVs: amplicon sequence variants
H site: highly exposed to asphalt
HC: highly exposed sediment without sulfate
HS: highly exposed sediment with sulfate
N site: non-exposed to asphalt
NC: non-exposed sediment without sulfate
NS: non-exposed sediment with sulfate
SRMs: sulfate-reducing microorganisms.
