# Supplementary material for: Sulfate Alters the Competition Among Microbiome Members of Sediments Chronically Exposed to Asphalt
Source: Front Microbiol. 2020 Sep 29;11:556793. doi: 10.3389/fmicb.2020.556793 (PMC7550536; doi:10.3389/fmicb.2020.556793)

SUPPLEMENTARY MATERIAL

Supplementary Table S1: Primer sets, thermal profiles and sources of positive standards used for real-time PCR and amplicon sequencing. Asterisks (*) indicate data collection steps.

Target gene	Primer set	Fragment length (bp)	Initial denaturation	Thermal profile	Number of cycles	Final elongation	Source of positive standard	Reference
**Real-time PCR**								
16S rRNA (bacteria)	FP 16S rDNA<br>RP 16S rDNA	263	95°C - 10 min	95°C - 45 s<br>58°C - 45 s<br>72°C - 45 s*	40	-	*P. putida*	Bach et al., 2002
16S rRNA (archaea)	rSAf(i)<br>958r	617	95°C - 10 min	95°C - 20 s<br>50°C - 1 min<br>72°C - 1 min*	5	-	*Methanobacterium* sp.	Uksa et al., 2015
				95°C - 20 s<br>50°C - 1 min<br>72°C - 1 min*	40			
dsrB	DSRp2060F<br>DSR4R	350	95°C - 10 min	95°C - 20 s<br>55°C - 20 s<br>72°C - 30 s*	40	-	*Desulfotomaculum* sp.	Geets et al., 2006
mcrA	mlas<br>mcrA-rev	470	95°C - 3.5 min	95°C - 30 s<br>55°C - 45 s<br>72°C - 30 s*	40	-	*Methanosarcinales* sp.	Steinberg and Regan, 2009
bamA	SP9<br>ASP1	300	94°C - 10 min	94°C - 30 s<br>59°C - 45 s<br>72°C - 1 min*	40	-	*G. metallireducens* GS-15	Kuntze et al., 2008
**Amplicon sequencing**								
16S rRNA (universal)	515FB<br>806RB	290	94°C - 3 min	94°C - 45 s<br>50°C - 1 min<br>72°C - 1.5 min	30	72°C - 10 min	*D. toluolica*	Apprill et al., 2015; Parada et al., 2016
dsrB	DSRp2060F<br>DSR4R	350	95°C - 10 min	95°C - 20 s<br>55°C - 20 s<br>72°C - 30 s	30	72°C - 10 min	*Desulfotomaculum* sp.	Geets et al., 2006
mcrA	mlas<br>mcrA-rev	470	95°C - 3.5 min	95°C - 30 s<br>55°C - 45 s<br>72°C - 30 s	30	72°C - 10 min	*Methanosarcinales* sp.	Steinberg and Regan, 2009

Supplementary Table S2: Statistical analysis on sulfate concentrations, gene abundances and Shannon diversities by robust tests. Test statistics (T, Q and F values), degrees of freedom (df) and p-values are shown for each test. Tested differences were considered significant when p-value was < 0.05 and marked with orange color.

		Statistical test	Independent variables		Sulfate	Genes: Bacteria 16S rRNA	Genes: Archaea 16S rRNA	Genes: Dissimilatory sulfate reduction *dsrB*	Genes: Methanogenesis *mcrA*	Genes: Degradation of monoaromatics *bamA*	Shannon diversity 16S rRNA	Shannon diversity *dsrB*	Shannon diversity *mcrA*
Day	0	robust t-test	Sediment	T	0.59	1.69	5.6	1.01	5.24	3.67	7.6	3.62	0.9
				df	3.66	8.57	9.82	9.08	8.24	9.1	9.61	6.69	5.09
				p-value	0.59	0.13	< 0.01	0.34	< 0.01	< 0.01	< 0.01	< 0.01	0.41
Sediment	NE	robust 2-way ANOVAs	Treatment	Qa	-	13.63	8.49	44.17	0.63	49.85	1.96	1.53	6.14
				p-value	-	< 0.01	< 0.01	< 0.01	0.44	< 0.01	0.19	0.23	0.04
			Day	Qb	-	24.79	74.9	92.05	243.96	49.93	0.58	50.55	71.13
				p-value	-	< 0.01	< 0.01	< 0.01	< 0.01	< 0.01	0.92	< 0.01	< 0.01
			Treatment:Day	Qab	-	3.52	0.95	24.16	7.22	5.83	0.37	6.97	17.37
				p-value	-	0.4	0.84	< 0.01	0.16	0.22	0.96	0.16	0.02
	HE	robust 2-way ANOVAs	Treatment	Qa	-	28.55	24.65	25.26	23.01	13.72	76.28	44.4	0.13
				p-value	-	< 0.01	< 0.01	< 0.01	< 0.01	< 0.01	< 0.01	< 0.01	0.73
			Day	Qb	-	1.5	9	20.07	5.39	3.48	179.31	98.07	92.27
				p-value	-	0.75	0.12	0.02	0.28	0.43	< 0.01	< 0.01	< 0.01
			Treatment:Day	Qab	-	19.06	10.5	26.27	11.29	9.09	64.02	18.66	5.7
				p-value	-	0.02	0.09	0.01	0.07	0.1	< 0.01	< 0.01	0.24
Condition	NC	robust 1-way ANOVAs	Day	F	-	4.29	11.2	10	42.41	3.7	0.15	21.08	20.33
				df1	-	3	3	3	3	3	3	3	3
				df2	-	6.5	6.25	6.53	5.94	5.44	5.84	6.41	6.03

				p-value	-	0.06	< 0.01	< 0.01	< 0.01	0.09	0.93	< 0.01	< 0.01
	NS	robust 1-way ANOVAs	Day	F	123.98	15.08	9.7	27	39.85	16.6	0.11	3.78	6.04
				df1	5	3	3	3	3	3	3	3	3
				df2	7.93	5.97	6.57	5.18	6.53	6.43	6.62	6.63	6.25
				p-value	< 0.01	< 0.01	< 0.01	< 0.01	< 0.01	< 0.01	0.95	0.07	0.03
	HC	robust 1-way ANOVAs	Day	F	-	7.92	6.22	4.3	5.95	6.78	12.68	6.43	11.71
				df1	-	3	3	3	3	3	3	3	3
				df2	-	5.92	5.25	5.91	5.49	6.49	5.73	3.06	4.73
				p-value	-	0.02	0.04	0.06	0.04	0.02	< 0.01	0.08	0.01
	Hs	robust 1-way ANOVAs	Day	F	21.4	1.5	0.08	6.12	0.22	0.19	56.71	75.42	14.68
				df1	5	3	3	3	3	3	3	3	3
				df2	7.51	5.36	5.42	5.01	5.82	6.64	6.37	5.23	6.33
				p-value	< 0.01	0.32	0.97	0.04	0.88	0.9	< 0.01	< 0.01	< 0.01

Supplementary Figure S1: Rarefaction curves of the sequenced libraries prepared for the genes of interest: (i) 16S rRNA, (ii) *dsrB* and (iii) *mcrA* genes. The number of ASVs detected in the datasets after rarefaction are presented as a function of the number of sequenced reads.

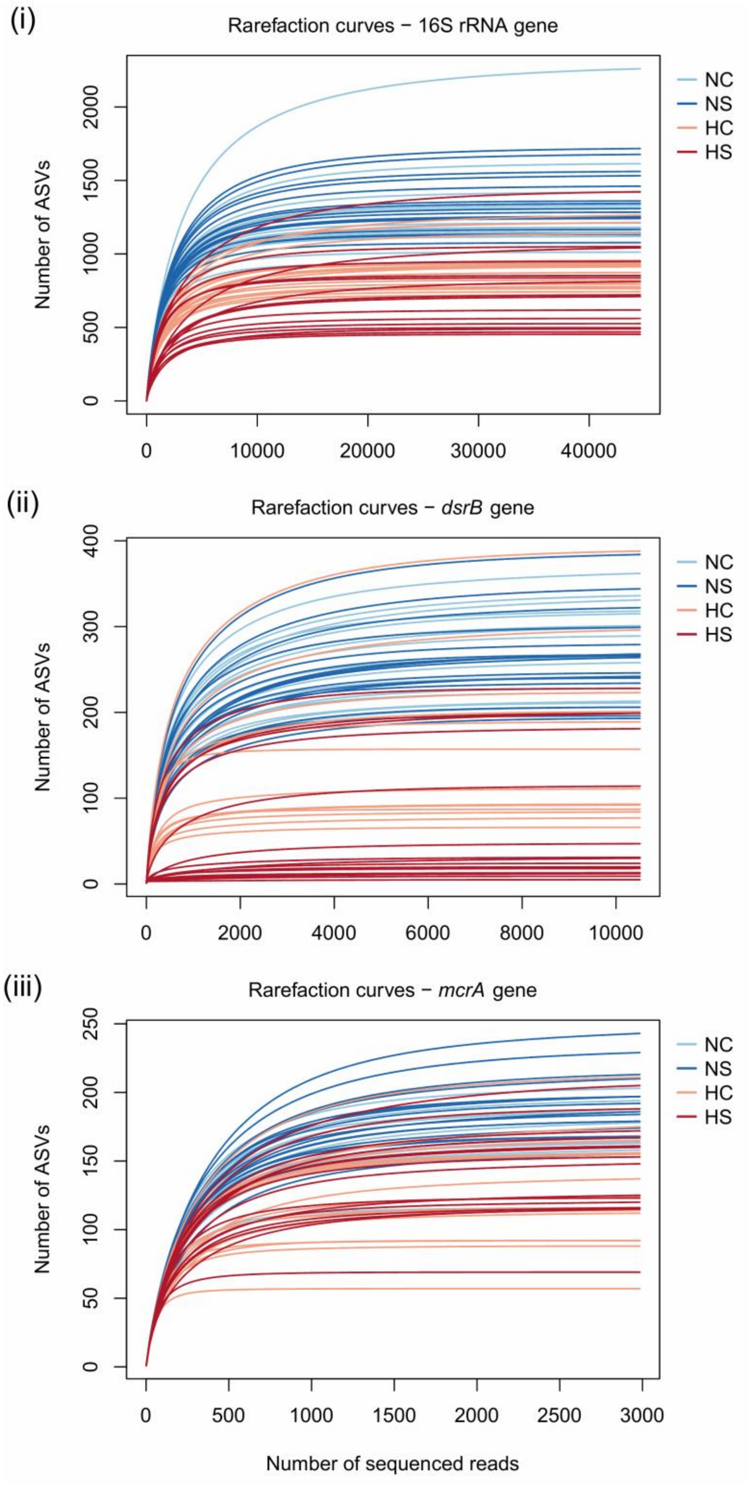

Supplementary Figure S2: Copy numbers of gene transcripts per g of dry anoxic sediment: bacterial 16S rRNA, archaeal 16S rRNA, *dsrB* and *mcrA*. The values are presented in square root transformed scale. Error bars indicate the standard deviation of the measurements in the four replicate microcosms. bl = all replicates below detection limit

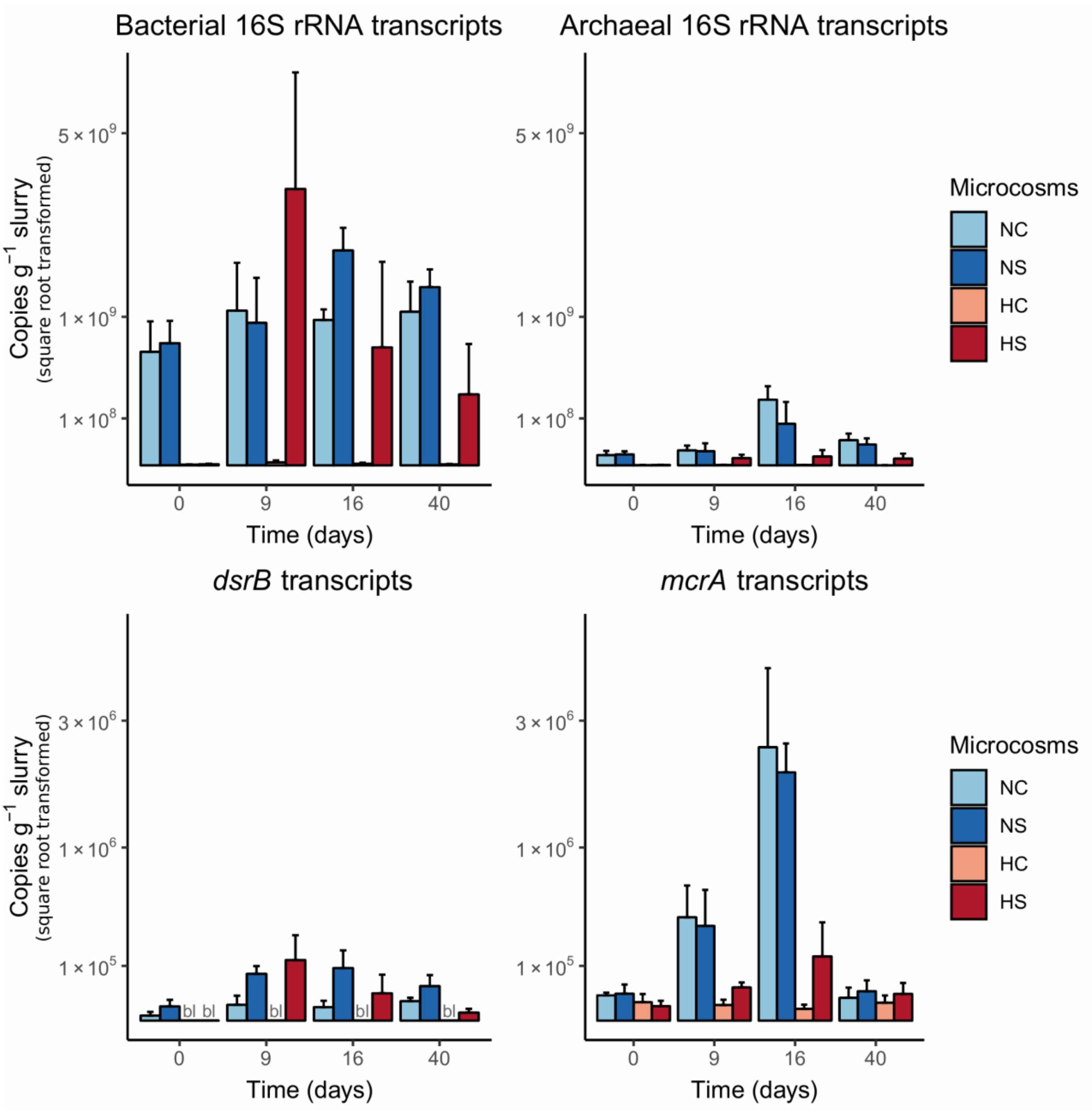

Supplementary Figure S3: Shannon diversity calculations based on the total ASVs of the sequenced 16S rRNA, *dsrB* and *mcrA* genes (top, middle and bottom panel, respectively). Error bars indicate the standard deviation of the measurements in the four replicate microcosms.

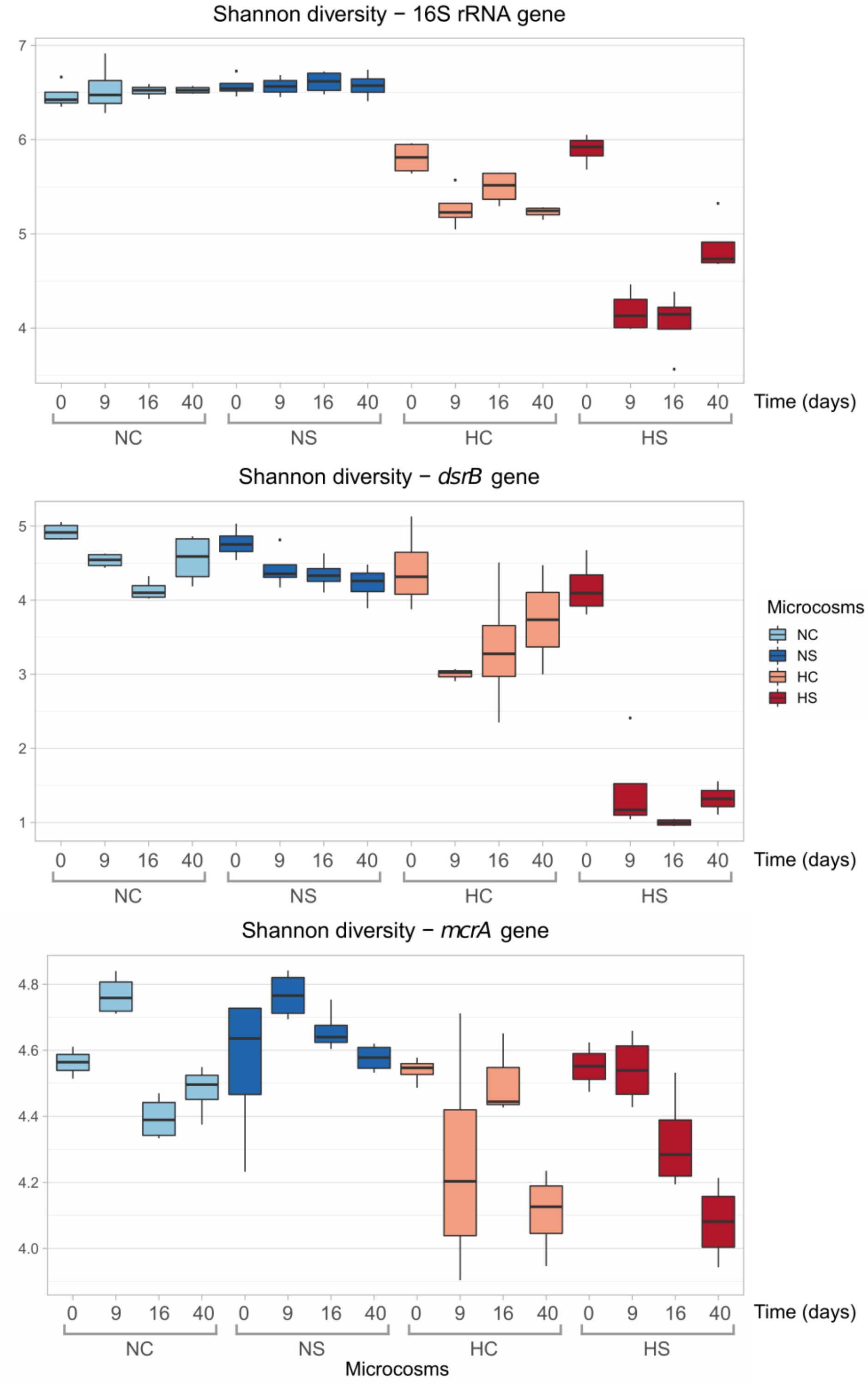

Supplementary Figure S4: Principal coordinates analysis (PCoA) of 16S rRNA gene libraries. Conditions are presented with different colors and sampling points with distinct shapes.

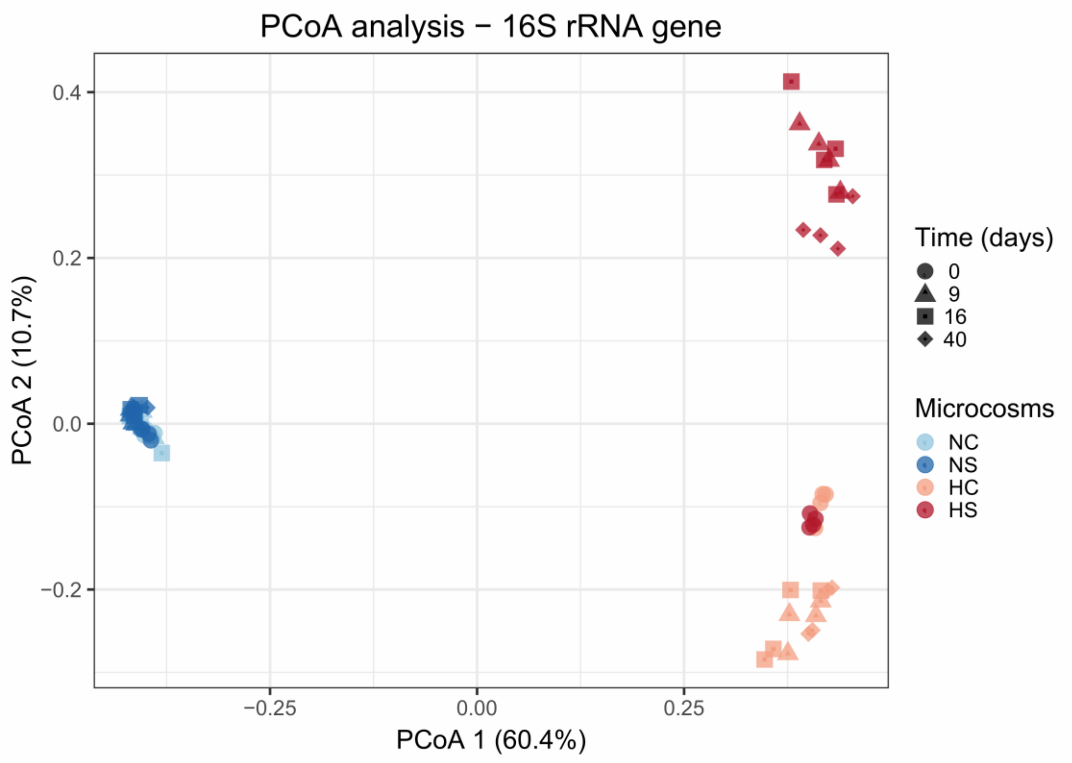

Supplementary Figure S5: Principal coordinates analysis (PCoA) of *dsrB* gene libraries. Conditions are presented with different colors and sampling time points with distinct shapes.

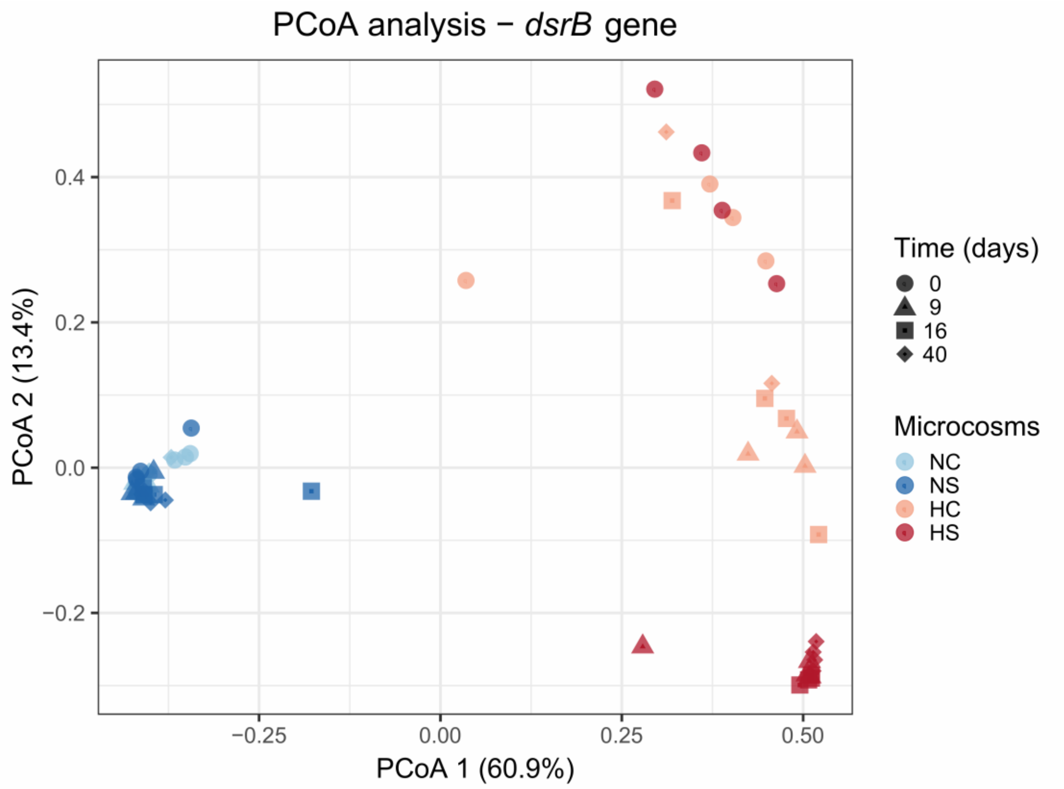

Supplementary Figure S6: Principal coordinates analysis (PCoA) of *mcrA* gene libraries. Conditions are presented with different colors and sampling time points with distinct shapes.

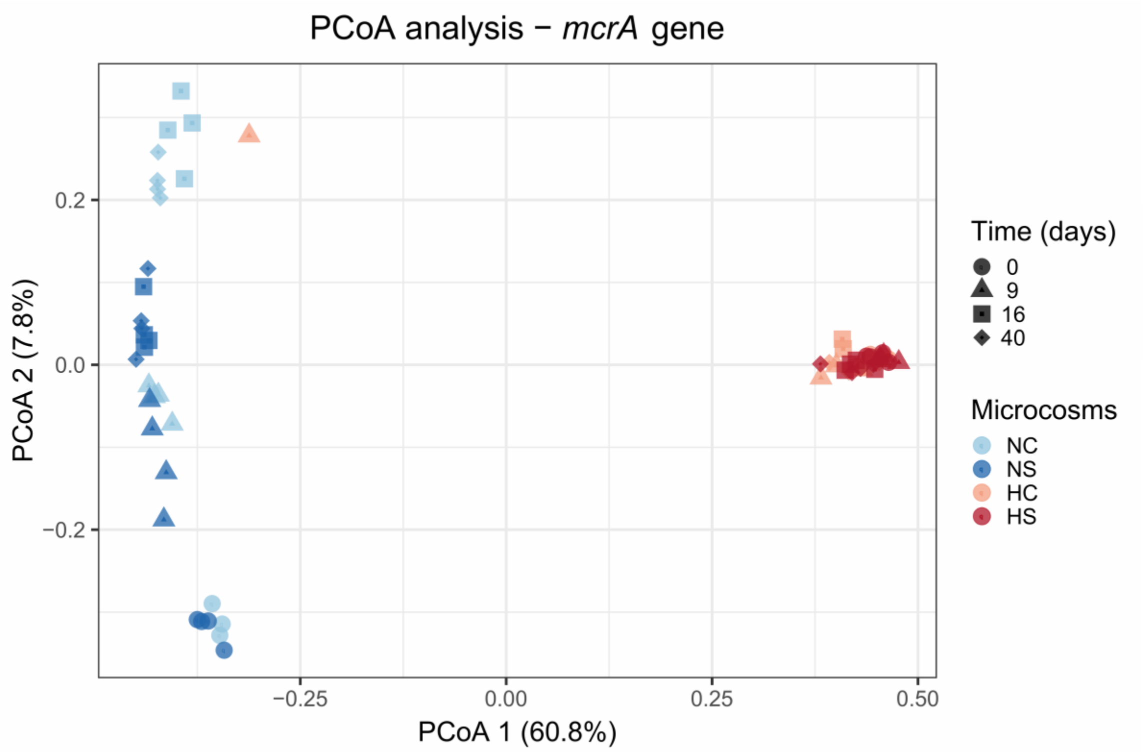

Supplementary Figure S7: Co-occurrence networks of the correlations in each condition. Bacterial and archaeal taxa are presented as white and black nodes, respectively. Bacterial taxa levels that include the genus *Desulfobulbus* (*Proteobacteria*, *Deltaproteobacteria*, *Desulfobacterales*, *Desulfobulbaceae*, *Desulfobulbus*) are marked with green in the sulfate conditions. The size of the nodes correlates to the abundance of the taxa and the thickness of the edges is proportional to the significance of the correlation. Positive and negative correlations are depicted with blue and red lines. Cc: clustering coefficient, cntr: centralization.

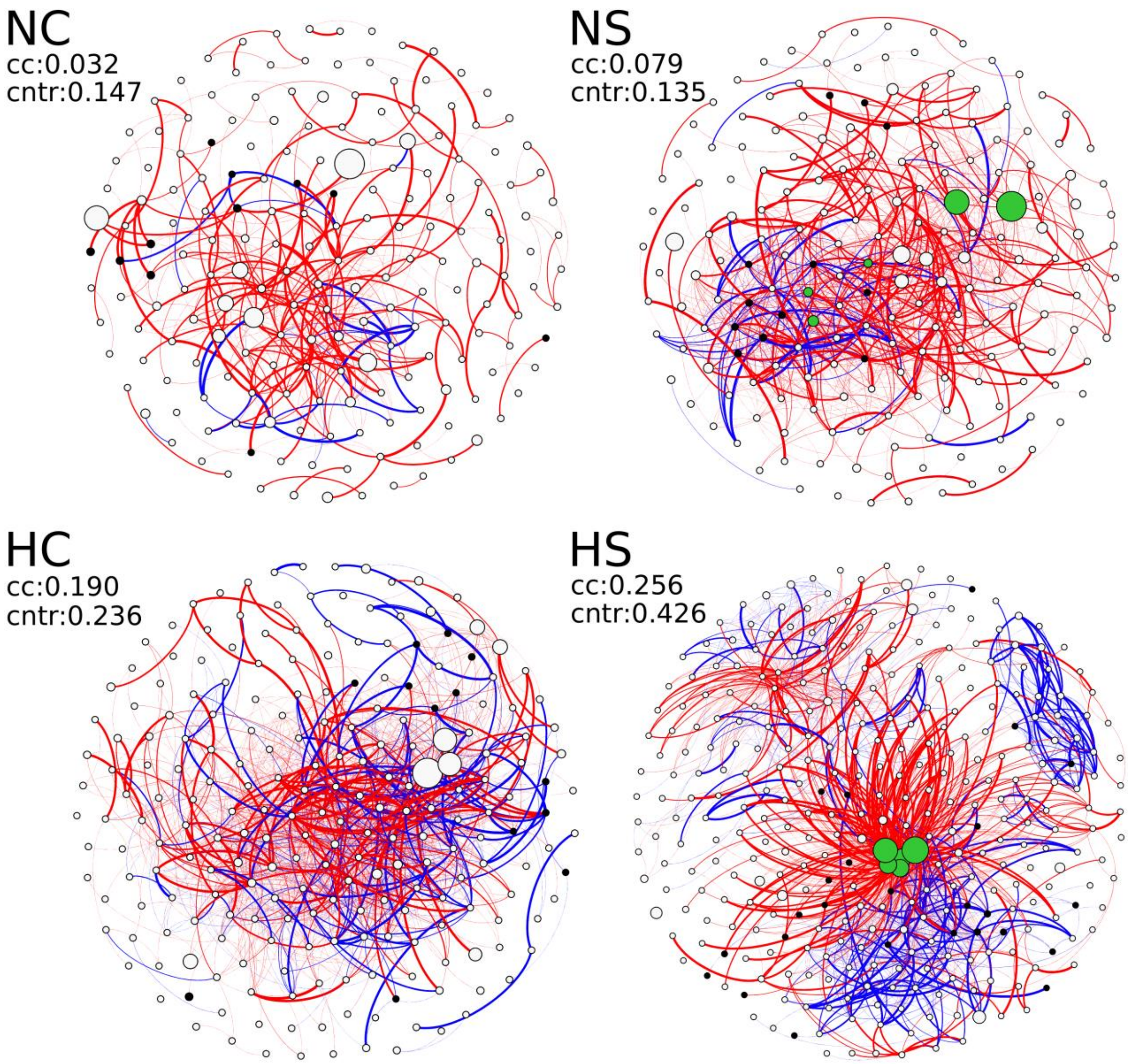

Supplementary Figure S8: van Krevelen diagrams of the different microcosms (color-coded composition: blue, CHO; orange, CHNO; green, CHOS; and red, CHNOS). Circular areas indicate relative mass peak intensity.

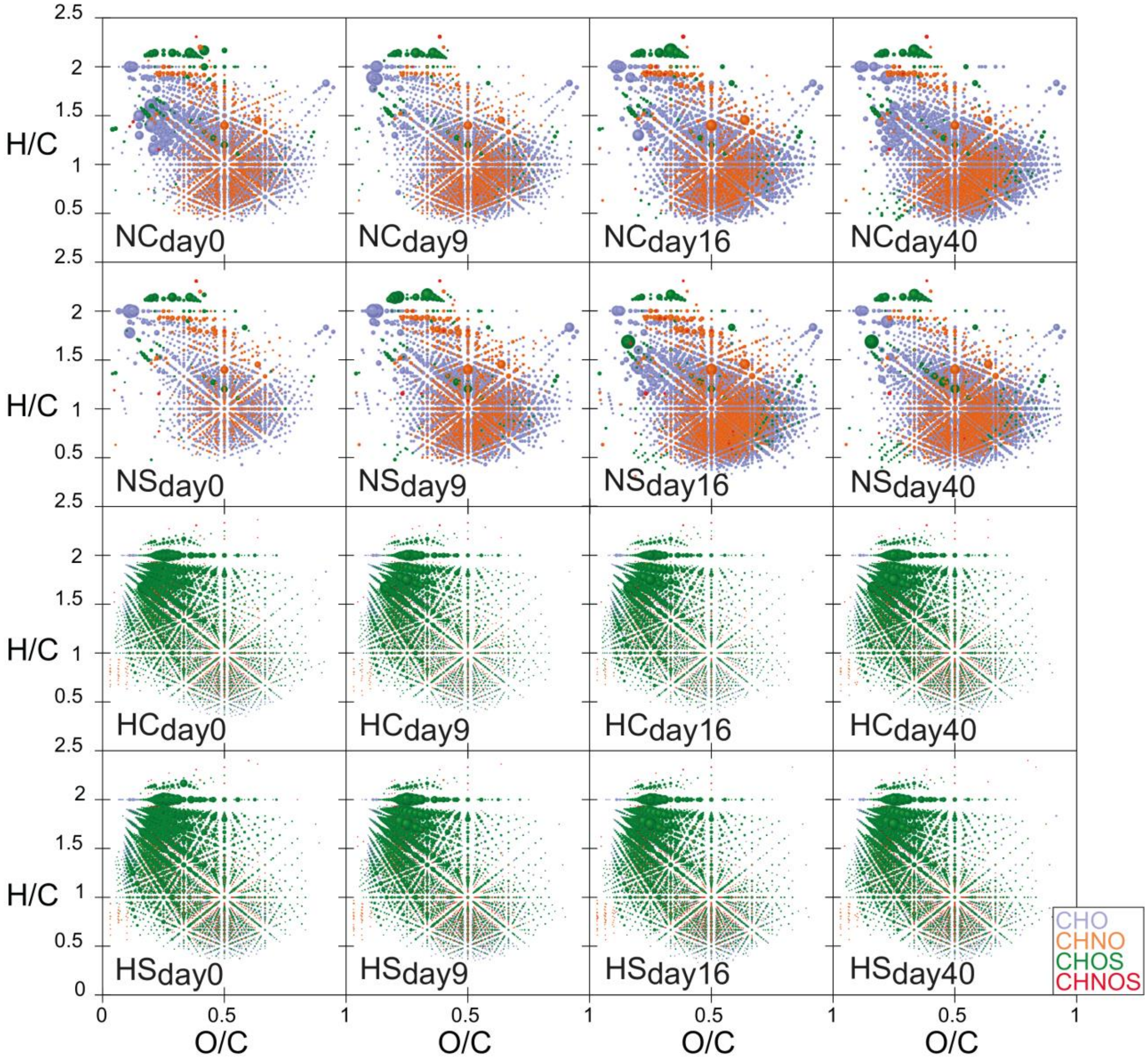

Supplementary Figure S9: Multilevel sPLSDA models based on FT-ICR MS data, which integrate the 16S rRNA (top panel) and *dsrB* gene data (bottom panel). For each component, the percentage of absorbed variation is reported.

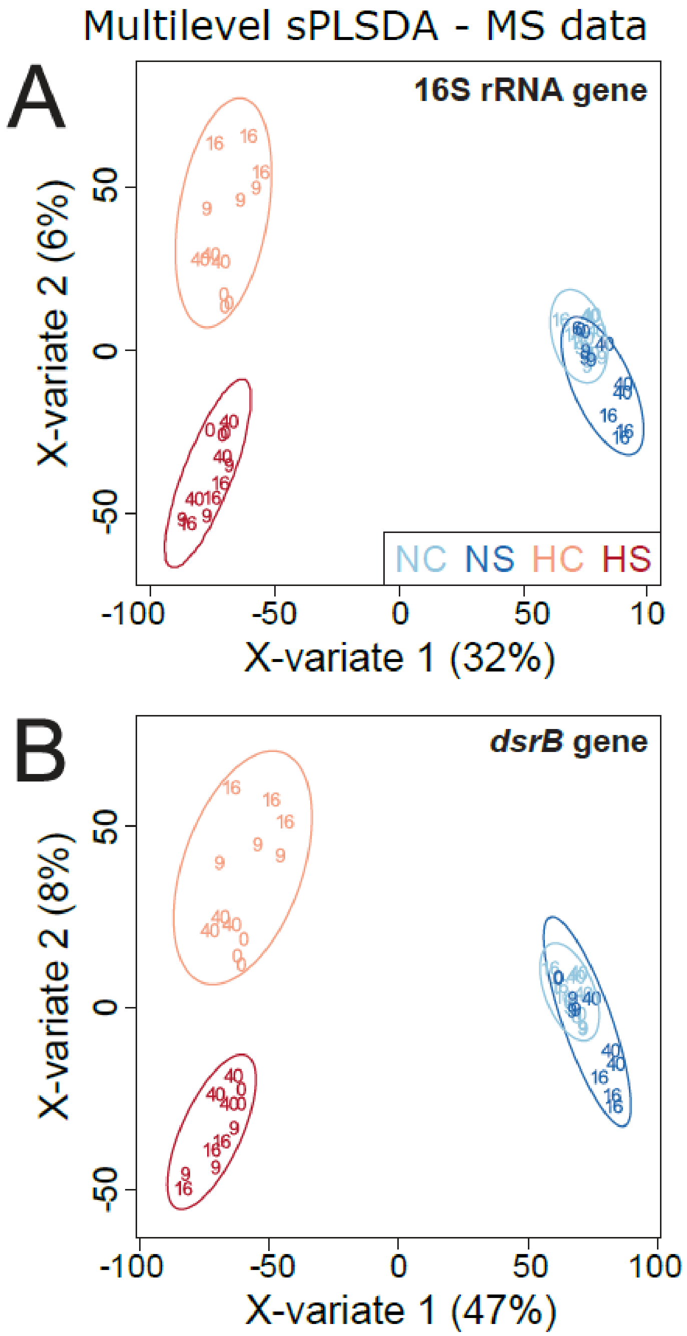

Supplementary Figure S10: van Krevelen diagrams of the most correlated m/z values based on the variables contribution on the first component. The diagrams visualize the chemical space of organic components in the microcosms containing highly-exposed and non-exposed sediment, respectively (see also Figure 7A). Color code for molecular series: blue, CHO; orange, CHNO; green, CHOS; and red, CHNOS.

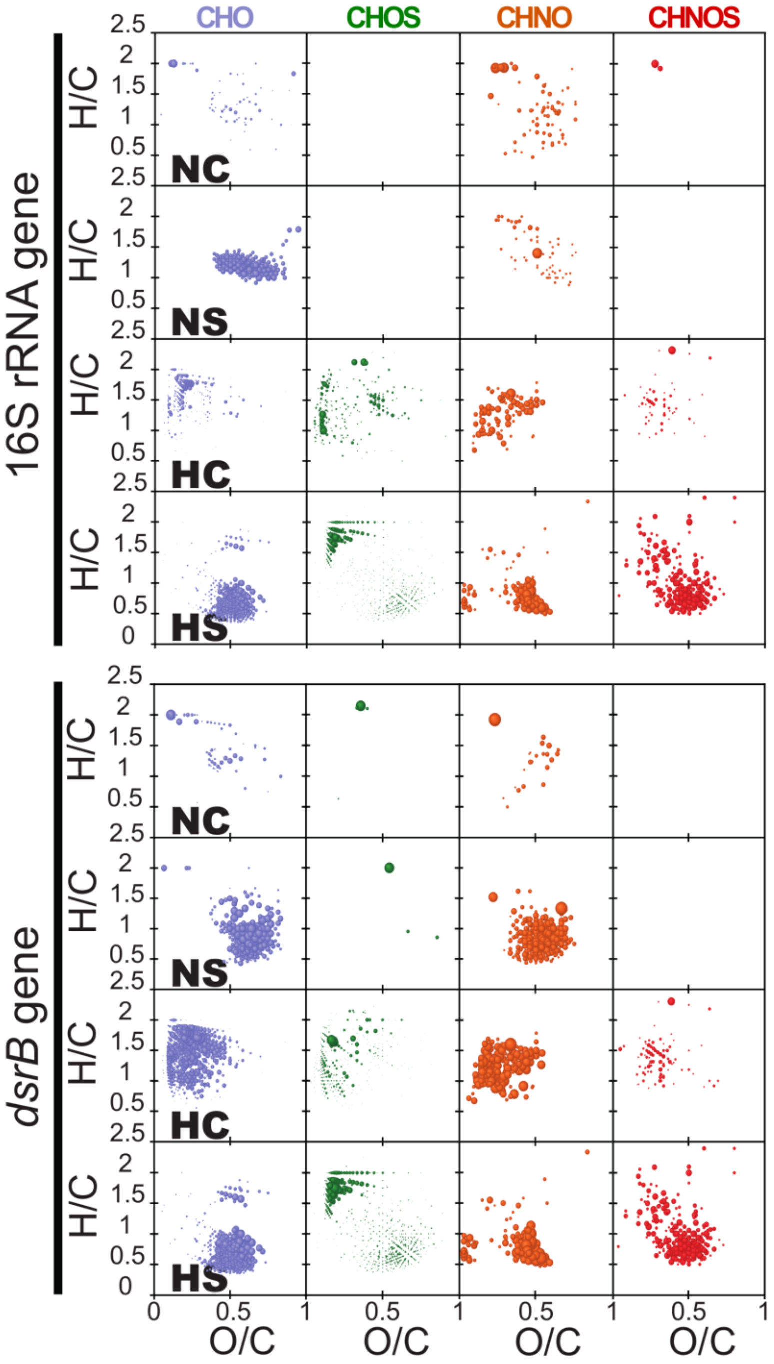

Supplementary Figure S11: Relative abundance (%) of the most abundant prokaryotic genera associated with changes in the abundance of the detected organic components.

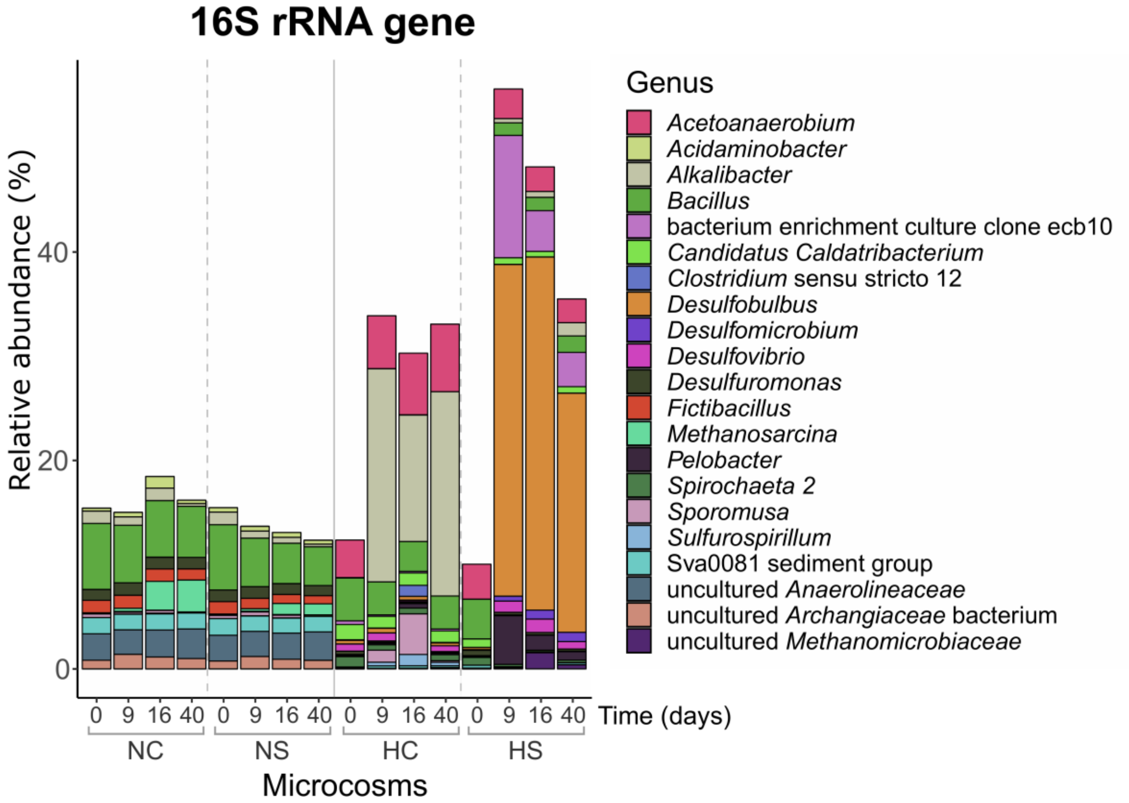

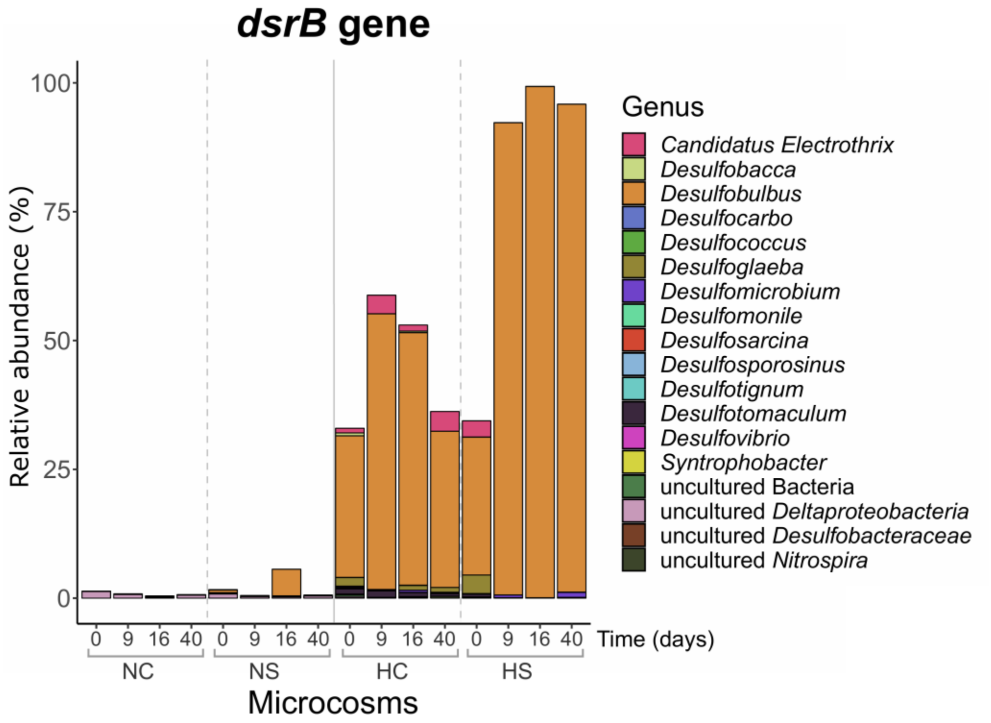

Supplement: Supplementary file 1 [file Data_Sheet_1.PDF]
